# Supplementary material for: Syphilis as Re-Emerging Disease, Antibiotic Resistance, and Vulnerable Population: Global Systematic Review and Meta-Analysis
Source: Pathogens. 2022 Dec 15;11(12):1546. doi: 10.3390/pathogens11121546 (PMC9785152; doi:10.3390/pathogens11121546)

Table S1 Newcastle-Ottawa Scale adapted for cross-sectional studies

Criteria	Condition required to obtain a start
Selection: 1) Representative of the sample (1 start)	Truly representative of the average in the target population, random sampling/ somewhat representative in the target population. non-random sampling
Selection: 2) Sample size (1 start)	Justified and satisfactory.
Selection: 3) Non-respondents (1 start)	Response rate is satisfactory (>60%). T. pallidum amplification rate
Selection: 4) Ascertainment of the exposure (2 starts)*	Use of biomarkers. molecular detection.
Compatibility: The subjects in different outcome groups are comparable, based on the study design or analysis (2 start)	Data/results adjusted to take into account relevant predictors/risk factors/confounders, e.g., age, sex, time since vaccination, etc
Outcome: 1) Assessment of the outcome (2 start)*	direct measurements using biomarkers are used. Molecular detection
Outcome: 2) Statistical test (1 start)	The measures (if any) including confidence intervals are detailed. In this case, the proportion of syphilis and mutation resistance.
This scale has been adapted from the Newcastle-Ottawa Quality Assessment Scale for cohort studies to perform a quality assessment of cross-sectional studies for the systematic review	
*In this study, greater emphasis was given on the detection of T. pallidum and antibiotic resistance, since the aim of this work was to describe and analyze the molecular detection of syphilis and mutations of the gene 23s RNAr T. pallidum	

Table S2. continuity of descriptive characteristics of eligible studies

First author, publication year	Specimen	Stage of syphilis
Bourgeois G, 2021 (20)	Lesions	Primary & secondary
Mitchell SJ, 2006 (21)	Lesions	Primary & secondary
Read P, 2014 (22)	Lesions, CSF, vitreous humor	Primary & secondary
Fernández-Naval C, 2019 (23)	Lesions, blood	Primary & secondary
Giacani L, 2018 (24)	Lesions	Primary & secondary
Nishiki S, 2020 (25)	Lesions, CSF, blood, tissue	Primary & secondary
Van Damme K, 2009 (26)	Lesions	Primary & secondary
Wu H, 2012 (27)	Lesions, CSF, blood, vitreous humor	Primary, secondary & neurosyphilis
Noda AA, 2016 (11)	Lesions	NR
Lu H, 2015 (28)	Lesions	Primary & secondary
Chen XS, 2013 (29)	Lesions, blood	Primary & secondary
Tipple C, 2011 (30)	Lesions, blood	Primary & secondary
Zhu B, 2016 (31)	Lesions	Primary & secondary
Martin IE, 2009 (32)	Lesions, blood, serum	Primary
Müller EE, 2012 (33)	Lesions	Secondary
Vrbová E, 2019 (34)	Lesions, blood	Primary & congenital
Liu D, 2021 (35)	Lesions, CSF	Primary, secondary & tertiary
Martin IE, 2009 (36)	Lesions, CSF, blood	Primary, secondary & congenital
Zondang HCA, 2020 (37)	Lesions	Primary
Katz KA, 2010 (38)	Lesions	Primary & secondary
Xiao Y, 2016 (39)	Lesions, blood	Secondary
Kanai M, 2019 (40)	Lesions, blood	Primary
Shuel M, 2018 (41)	Lesions, CSF, blood	Primary, secondary & congenital
Mikalová L, 2017 (42)	Blood, CSF	Primary, secondary & neurosyphilis
Grillová L, 2014 (43)	Lesions, CSF, blood, tissue	Primary, secondary & congenital
Gallo Vaulet L, 2017 (44)	Lesions, CSF, blood	Primary, secondary & congenital
Martin IE, 2010 (45)	Lesions, CSF, blood, vitreous humor	Primary, secondary & congenital
Wu BR, 2014 (46)	CSF, serum, plasma, vitreous humor	Primary, secondary & neurosyphilis
Grillová L, 2019 (47)	Lesions	Primary
Xiao H, 2017 (48)	Lesions	Primary & secondary
Su JR, 2012 (49)	Lesions	Primary & secondary
Flasarová M, 2012 (50)	Lesions, CSF, blood, knee aspirate, tissue	Primary, secondary, tertiary & congenital
Sanchez A, 2020 (51)	Lesions	Primary & secondary
Wu BR, 2014 (52)	CSF, serum, plasma, vitreous humor	Primary, secondary & neurosyphilis
Khairullin R, 2016 (53)	Lesions, blood	Primary & secondary
Muldoon EG, 2012 (54)	Lesions, CSF, blood, tissue, vitreous humor	Primary, secondary & neurosyphilis
Coelho EC, 2021 (55)	Lesions	Primary & secondary
Venter JME, 2021 (56)	Lesions, blood	Primary & secondary
Flores JA, 2016 (57)	Lesions, serum	NR
Li Z, 2013 (58)	Lesions	Primary & secondary
Grimes M, 2012 (59)	Blood, CSF	Primary & secondary

General characteristics of eligible studies NR: Not reported; CFS: cerebral spinal fluid

Figure S1 Meta-analysis of proportion global of molecular detection of syphilis.

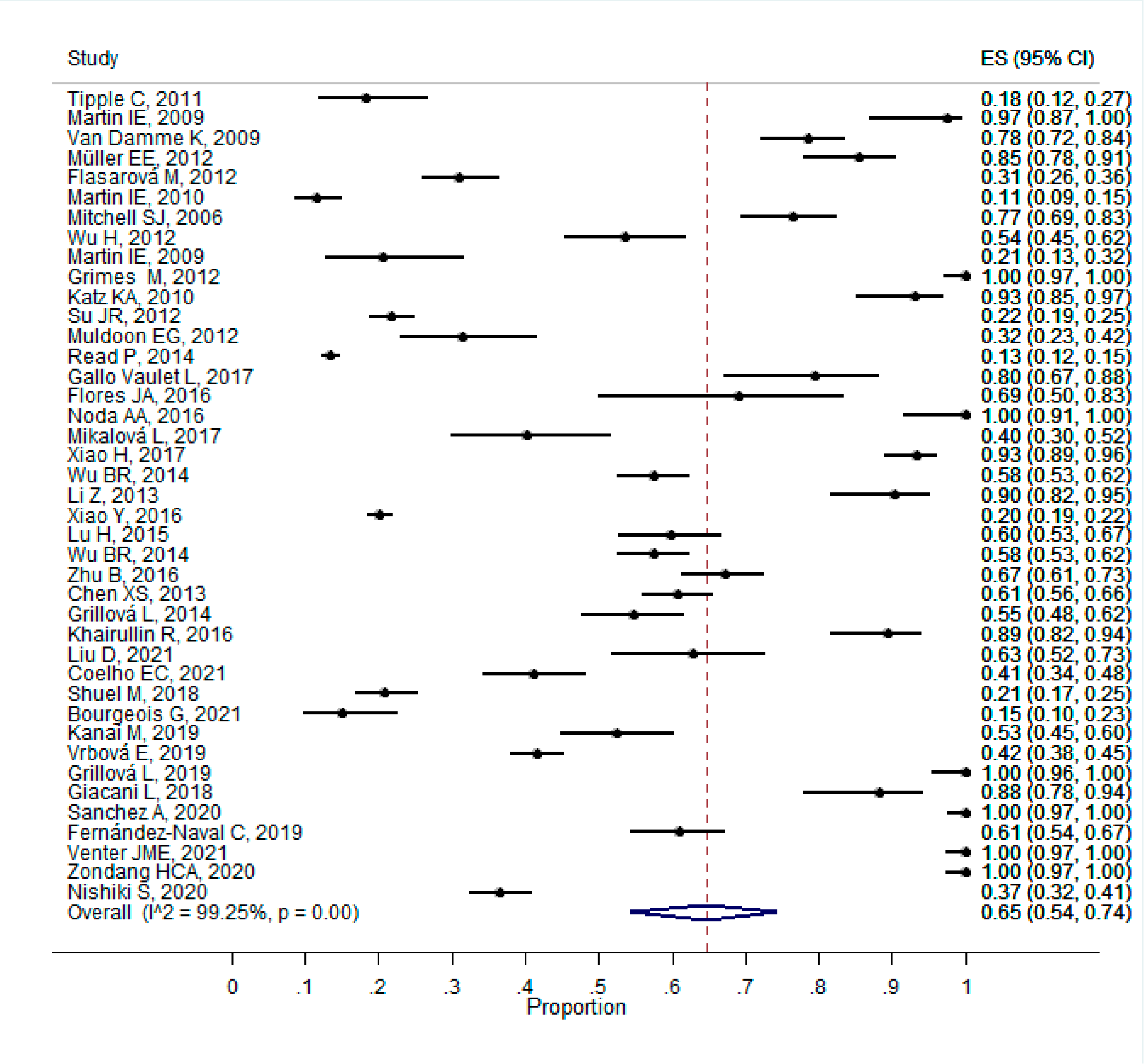

Figure S2. Meta-analysis of the proportion of A2058G mutations of the eligible studies

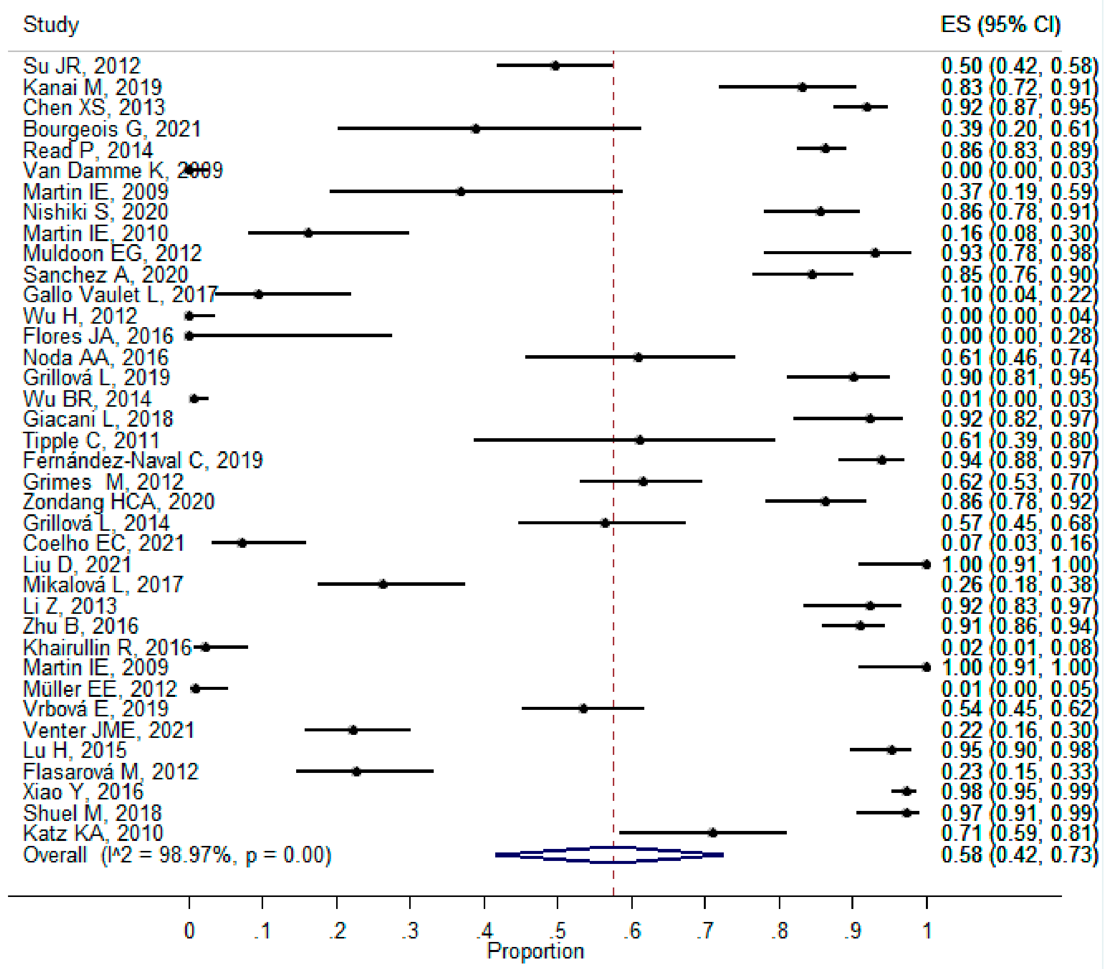

Supplement: Supplementary file 1 [file pathogens-11-01546-s001.zip › pathogens-2046121-supplementary.pdf]
